# Do conservatives really have better mental well-being than liberals?

**DOI:** 10.1371/journal.pone.0321573

**Published:** 2025-04-30

**Authors:** Brian F. Schaffner, Thomas Hershewe, Zoe Kava, Jael Strell

**Affiliations:** 1 Department of Political Science/Tufts University, Medford, Massachusetts, United States of America; 2 Department of Political Science/Yale University, New Haven, Connecticut, United States of America; Universite de Sherbrooke, CANADA

## Abstract

American conservatives tend to rate their mental health more positively than their liberal counterparts. One explanation for this finding is that conservatives may be more likely to justify existing inequalities in society, leading to a palliative effect on their mental health that does not happen for liberals. Conservatives also score higher on personality and attitude measures, such as religiosity, marital status, and patriotism, which are associated with better mental health. We examine whether this ideological mental health gap holds for a different facet of well-being that is closely related to mental health. Further, we suggest that the ideological mental health gap may have more to do with a stigmatized reaction to the term “mental health” which has become increasingly politicized in the US context since its introduction to literature in the early 20th century. First, we examine whether the conservative-liberal divide in self-assessments of mental health remains once we control for a wide variety of demographics, socioeconomic factors, and recent life experiences. We find that accounting for these alternative explanations reduces the gap by about 40%, but that ideology remains a strong predictor of mental health self-reports. Second, we conducted an experiment where we randomly assigned whether people were asked to evaluate their mental health or their overall mood. While conservatives report much higher mental health ratings, asking instead about overall mood eliminated the gap between liberals and conservatives. One explanation is that rather than a genuine mental health divide, conservatives may inflate their mental health ratings when asked, due to stigma surrounding the term. Another possibility is that ideological differences persist for some aspects of mental well-being, but not others.

## Introduction

According to a number of studies, conservatives are happier and have better mental health than liberals [1–11]. A 2006 public opinion survey found that 47% of Republicans in the United States said they were “very happy” compared to only 28% of Democrats [12]. Since 1972, conservatives have consistently reported greater levels of happiness compared to liberals [8]. This ideological gap in happiness exists in the United States but there is inconsistent evidence for its presence in other countries [7, 10]. These findings have received widespread attention in news outlets with headlines such as “How the right turned radical and the left became depressed” and “Are American progressives making themselves sad?”[13, 14]

One explanation for this ideological gap in mental health could stem from differing ideological justifications for the state of the world. Conservatism is a system-justifying ideology that seeks to rationalize the existing political, economic, and social order [3, 5]. On one hand, conservatives rationalize inequality in the world to justify their privilege within the system they are living in, ultimately serving as a buffer against the negativity of inequality and preserving a positive mental state [5, 15–17]. Liberals, on the other hand, are less inclined to rationalize inequality in this way, recognizing that there are factors outside of one’s control that affect outcomes, and that hard work alone may not be enough to achieve a positive outcome [9]. The belief that life events have significance and direction was also linked to less negative affect during the COVID-19 pandemic [18]. On the other hand, perceived futility could have a net negative effect on mental health. As inequality increases, people report worse mental health. And since liberals lack an ideological rationalization which frames inequality in a positive or neutral light, the existence of inequalities takes a greater toll on their mental state [5, 17, 19].

Conservatives’ greater levels of justifications for and acceptance of the current state of the world (e.g. inequality) serve as a pacifying factor for their own mental state. If the conservative ideology allows for this system justification – an acceptance of the status quo – while the liberal ideology does not, then it might help explain why conservatives have better mental health. And this pattern may be cyclical as conservatism may prompt healthy, positive responses to adversity, while liberalism may lead individuals to respond to hardship in ways which are detrimental to their well-being [20–22]. In a longitudinal study measuring adolescent mental health from 2005 to 2018, all groups’ mental health trended downward regardless of political identity, However, starting in approximately 2010, female liberal adolescents had the largest, most significant negative change in depressive affect, self-esteem, self-derogation, and loneliness [3]. This drop coincides with the popularization of the cell phone and the proliferation of digital news that defines the current political landscape. There is the potential that these developments have helped intensify the effect of political beliefs and other demographic factors on mental health in adolescents [3].

However, there are at least two reasons to question whether there is something inherent in political ideology that produces genuine differences in people’s mental health. First, it may be that factors correlated with ideology are actually responsible for the mental health gap rather than ideology itself being the culprit. Conservatism is positively correlated with a number of traits that are traditionally associated with better mental health and well-being, such as religious faith, patriotism, marriage, higher incomes, and old age [1, 4, 9, 18, 20, 23–26]. To provide one example, religion can provide a source of social support, a purpose in life, positive life choices, a coping mechanism for when things go wrong [23, 26]. It is also true that conservatives in the United States are more likely to be religious than liberals. Thus, at least some portion of the ideological mental health gap may actually be caused by religiosity.

Under this view, conservatives are happier than liberals because conservatism is associated with numerous factors that complement a more positive psyche. In addition to being more religious, conservative Americans are also more likely to get (and stay) married, more likely to be financially secure, and tend to be older. Each of these factors also tends to be associated with positive mental health. Likewise, liberal Americans are younger, less likely to be religious, more likely to be members of socially ostracized groups, and less likely to marry. Liberals are also more engaged in politics, participate more, and more likely to find meaning in political activism, but involvement in politics appears to have a negative impact on well-being [27, 28]. Among less advantaged groups, such as those with a lower socioeconomic status, conservatism has not been linked to improved mental health. Instead, individuals see their lower status as a personal failure, heightening negative perceptions of self [3].

If the ideological mental health gap is at least partially explained by factors associated with ideology rather than by ideology itself, then a more accurate estimate of the size of this gap requires controlling for such factors. While many studies have attempted to do this, the scope of variables and the size of the sample collected must be sufficiently large to produce an exhaustive accounting of these alternative explanations. In Study 1 we provide just such an analysis by using a representative survey of 60,000 American adults with a sufficient breadth of questions to control for more than two dozen potentially confounding variables. As we show, about 40% of the ideological gap in mental health self-assessments is attributable to these compositional effects.

However, even controlling for a wide variety of alternative factors the ideological mental health gap still persists. This leads to a potential alternative reason for this gap: the possibility that liberals and conservatives may respond very differently to the survey questions used to establish this gap. Notably, much of the research examining the mental health or happiness gap relies on self-reported measures of subjective well-being (or even self-reports of mental health diagnoses). For instance, respondents in a survey are often asked to rate their own mental health based on a subjective scale. But it may be the case that liberals and conservatives do not answer these questions in equally honest ways. For example, conservatives may be more likely to self-enhance when answering surveys, leading them to provide more positive assessments of their mental health than they actually feel internally [11, 29].

This tendency is exacerbated by the fact that the concept of mental health is often stigmatized by conservatives. While mental health is in part a scientific concept, it has also become politicized [30]. The literature on stigma and ideology is suggestive of a strong association between right-wing authoritarianism, which relates to conservative ideology, and the stigmatization of mental health, such as holding negative stereotypes and distancing themselves from those with mental illnesses [31–35]. Many mental illnesses often are stereotyped as dangerous, unpredictable, indicative of bad character, or a sign of personal weakness [36–44]. Under a conservative ideology that emphasizes stability, security, and personal agency, the thought of mental illness may invite thoughts of the unknown or appear as a threat to their security. From a personal agency perspective, struggling with mental health such as depression would be viewed as someone’s own doing and not working hard enough to overcome their struggles. Here, stigma serves as a strategy to protect one’s personal values. That is, if people with mental illness are responsible for their illness, they are able to preserve their view of the world [35]. If conservatives associate poor mental health with notions of danger or weakness, they may inflate their own mental health ratings to avoid confronting a negative image of themselves.

Thus, rather than a genuine difference between liberals and conservatives, the mental health gap may reflect conservatives’ tendency to make themselves appear better on surveys. There is some evidence to support this proposition. For instance, while conservatives report greater happiness, liberals may be more likely to demonstrate signs of happiness: liberal politicians expressed more positive emotional language as well as smiled more intensely and genuinely, and liberal Twitter users were more likely to tweet out positive words and messages than conservatives on Twitter [11].

If conservatives and liberals engage with the term “mental health” in ways that lead them to differ in the veracity of self-assessments they provide in surveys, then what is needed is a term that is less stigmatized by conservatives. For this reason, our second study is an experiment fielded on a sample of 1,000 American adults in which we randomly assigned each respondent to provide a self-assessment of either their mental health or their overall mood.

Of course, it is important to recognize that one’s mental well-being consists of multiple overlapping but distinct dimensions, including mental health and overall mood. The existing research addresses happiness, subjective well-being, mental health, or mental illness [9, 47]. While each of these terms are similar and related, they do not all come with the same meaning. We indeed recognize that the terms “mental health” and “overall mood” are not synonymous. Mental health encapsulates a range of behaviors and symptoms that mood does not necessarily capture. Mental health tends to represent a more long-term state of well-being, whereas an individual’s overall mood is a more short-term state.

It is possible, then, that overall mood, given its shorter term nature, can be influenced by daily stressors and emotional fluctuations that are less tied to ideology. For example, liberals and conservatives might react similarly to a negative life event such as a death in the family. Asking an individual about their “overall mood” might capture a more immediate response to an individual’s life events, or current state of being. “Mental health”, given its longer term nature, might reflect an individual’s ability to cope with these daily stressors. A person said to be in “good mental health” may be sad, angry, or upset but be well-equipped to deal with these emotions [48]. It is possible that conservatives have better mental health than liberals but not overall mood reflecting the fact that they are better able to cope, in a longer term sense, with daily stressors or negative life events.

While we find a significant and robust gap in mental health ratings between liberals and conservatives, we also find that this difference disappears when we ask about overall mood instead. Based on the discussion above, we suggest that there are two possible interpretations of this difference. The first is that ideology is simply not a predictor of one’s overall mood in the way that it is for their mental health. The second possible explanation is that right-wing stigma behind the language of “mental health” leads respondents to inflate their mental health ratings in a way that they do not when asked about “overall mood.”

## Methods

Our data comes from the 2022 and 2023 Cooperative Election Study (CES) surveys. The CES is an NSF-funded survey about politics that is fielded each year. The surveys are carried out online by the firm YouGov using nationally representative samples of American adults. All analyses presented in the report use the weights provided by YouGov to ensure that the estimates apply to a representative sample of adults. Extensive detail about the methodology of the CES survey can be found in the guide to the CES, located here: doi: 10.7910/DVN/PR4L8P.

We used the 2022 CES Common Content to examine the conservative-liberal divide in self assessments of mental health; we used a 2023 CES team module (sample subset) to field our experiment. Both surveys were reviewed by Human Subjects office and ruled exempt (2022: Harvard University IRB19-1411; 2023: Tufts University STUDY00004327). For the 2022 CES, responses were collected from September 29th to November 8th. For the 2023 CES, responses were collected from November 8th to December 11th. For both surveys, respondents first provided their informed consent before moving forward in the surveys.

## Study 1

To measure mental health self-assessments, respondents were asked: “Would you say that in general your mental health is...” with five responses offered: Excellent, Very Good, Good, Fair, and Poor. For Study 1, of the 60,000 respondents to the 2022 CES, 59,710 responded to this question with the remaining 290 respondents (approximately 0.5% of the sample) choosing to skip it.

To identify the association between ideology and mental health self-assessments, we used a question asking respondents to identify their own ideological leanings. Specifically, this question asks respondents how they would rate themselves on a scale with seven options: very liberal, liberal, somewhat liberal, middle of the road, somewhat conservative, conservative, and very conservative. Respondents were also provided with the option of answering “not sure.” We re-scaled this variable to range from 0 to 1, with very liberal respondents coded as zero and very conservative respondents coded as 1 and the remaining response categories distributed equidistant between those two end-points. We note that the use of an ideological self-identification question means that we are capturing symbolic ideology – how people self-identify ideologically – rather than operational ideology – the ideological structure to their issues attitudes [49]. This approach is consistent with much of the existing scholarship on ideology and mental health [5, 9, 18]. Nevertheless, in the Supporting Information (Table S3 we reproduce the results from Study 1 using a measure of operational ideology and show that our results are consistent.

The main analysis for Study 1 is a regression model predicting a respondent’s self-reported mental health on a 0 to 1 scale with 0 representing those who identified their mental health as “poor” and 1 for those who said it was “excellent.” The remaining response categories are distributed equidistant apart between those end-points.

In addition to a variable capturing each respondent’s ideological self-placement, we also included a wide range of additional variables. Demographic and socioeconomic variables included age, race, education, marital status, parental status, employment status, type of community in which the individual resides, and frequency of church attendance. We included a set of items to capture economic well-being, including income, whether the respondents owns a home, whether the respondent owns stocks, and whether the respondent would be unable to cover an unexpected $400 expense without going into debt. The latter item is frequently used by the Federal Reserve to measure economic vulnerability [50]. We also included variables capturing notable events that the respondent reported having experienced during the past year, including having gotten married, gone through a divorce, had a child, went to the doctor, visited the emergency room, was the victim of a crime, finished school, retired, lost a job, got a new job, got a pay raise, got a pay cut, had Covid-19, gotten vaccinated for Covid-19, and moved. Finally, we included two variables capturing media consumption – whether the respondent uses social media and whether the respondent reports following news about public affairs most of the time.

All of the questions used in our analysis were included on the pre-election wave of the 2022 CES, which was fielded from September 29th - November 8th.

**Fig 1:**
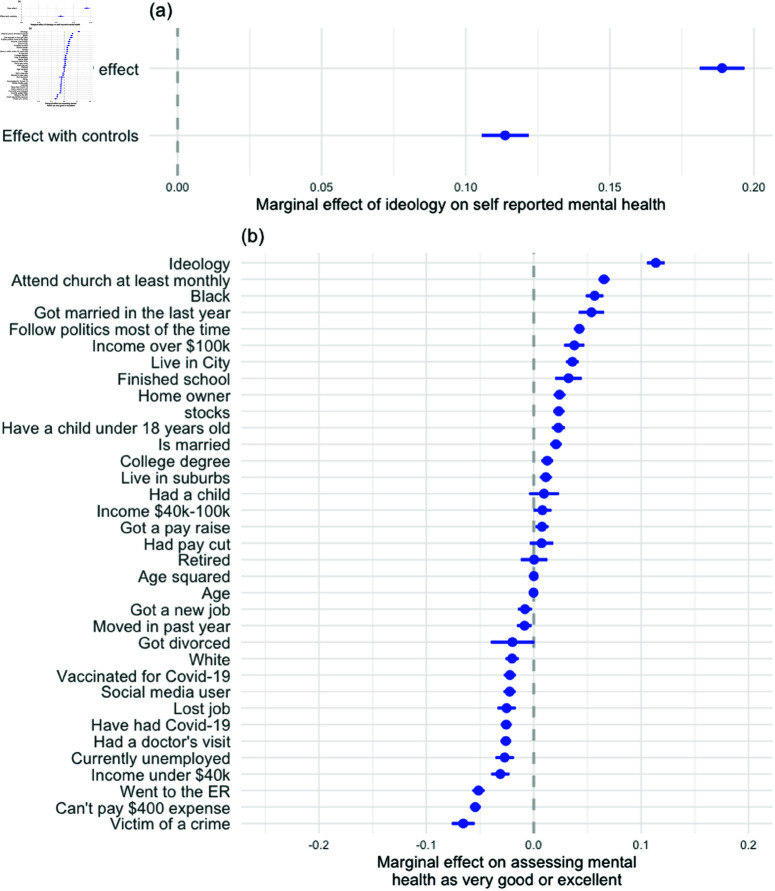
Modeling self-assessments of mental health in the 2022 Cooperative Election Study survey. (**a**) Coefficient for the ideology variable in a model with no control variables compared to a model with all control variables included. (**b**) Coefficient for each independent variable. Horizontal lines represent 95% confidence intervals.

Full model results for Figure 1 are available in the Supplementary Information Table S1 as well as an additional analysis that separately analyzes responses to the most positive and most negative self-assessment categories (Table S2).

## Study 2

The experiment (study 2) was fielded on a module (sample subset) of the 2023 CES. The module was administered to 1,000 American adult respondents. Each respondent was randomly assigned to one of two conditions, with 500 respondents in each condition. Respondents in the first condition were prompted with the same mental health assessment question and response options from the 2022 survey (Study 1). The other half of the sample saw the same question wording except the term “overall mood” was exchanged for “mental health.”

## Results

We conducted two studies – the first involves determining the size of the mental health gap after controlling for a large number of alternative explanations and the second is an experiment testing whether the gap disappears when we ask instead about a respondent’s overall mood. We discuss the results from each in turn.

### Study 1: Accounting for compositional effects

Our first study uses a survey of 60,000 American adults to test for the conservative-liberal divide in self-assessments of mental health and to test how much of this divide remains once we control for a wide variety of demographics, socioeconomic factors, and recent life experiences. The very large sample size and breadth of information collected on respondents allows us to account for many factors that might be correlated with ideology, but have their own independent effect on mental health self-assessments.

Figure 1 presents the results from this analysis. Panel (a) summarizes how the marginal effect of ideology is affected by controlling for a host of variables. Panel (a) shows that moving the full length of the ideological scale (from very liberal to very conservative) is associated with about a 19-point increase in reporting positive mental health. However, at least part of this gap is due to compositional effects, such as the fact that conservatives tend to be older and are more likely to attend church. Once we account for the wide range of other factors, the gap is reduced to 11 percentage points, about 60% of its original size.

Panel (b) plots the effects of each variable we included in our regression model. While the size of the ideological gap was significantly reduced by controlling for other factors, ideology was still the strongest predictor of how people assessed their own mental health, alongside traits like being older, being African American[51], and attending church frequently. The factors most strongly associated with a negative effect on mental health self-assessments include having been the victim of a crime during the past year, having visited the emergency room during the past year, and being unable to cover an unexpected $400 expense without going into debt.

Overall, the results from Study 1 show that accounting for other factors correlated with ideology significantly reduces but does not eliminate the mental health gap between conservatives and liberals.

### Study 2: Experimental test using alternative terminology

**Fig 2:**
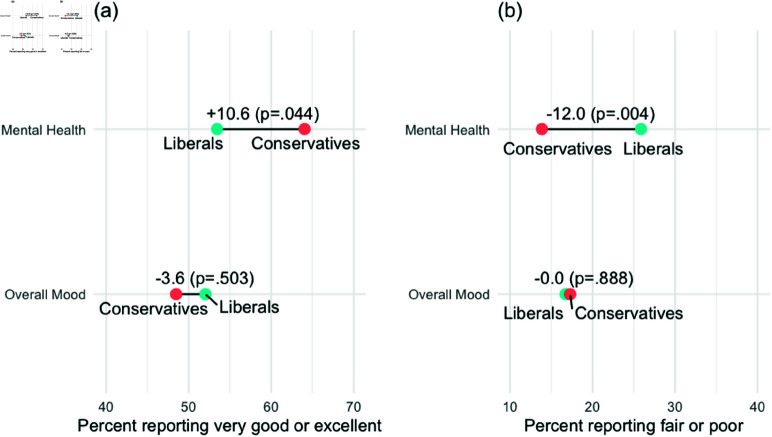
Effects of experimental condition on the Conservative - Liberal gap in self-assessments of mental health vs. overall mood.

In our second study, we employed an experiment dividing our sample in half and exchanging the term “mental health” with “overall mood” for one half of the sample (randomly assigned). Figure 2 shows how this wording alteration affects the gap between liberals and conservatives when it comes to giving highly positive self-assessments (panel a) and negative self-assessments (panel b). When respondents were asked to assess their own mental health, conservatives were almost 11 points more likely to respond “excellent” or “very good” as compared to liberals (p = 0.044). Conservatives were 12 points less likely to select “fair” or “poor” when compared to liberals (p = 0.004).

When respondents were asked to assess their overall mood, the gap between liberals and conservatives disappeared. Conservatives were actually somewhat less likely than liberals to provide a highly positive rating of their overall mood, though this difference was not statistically significant (p = 0.225). And the gap between conservatives and liberals on selecting “fair” or “poor” overall mood was nearly zero (p = 0.888).

The figure also reveals that the reason for the elimination of the gap on either end of the scale comes from different sources. For highly positive ratings, the movement comes almost entirely from conservatives, 64% of whom gave a highly positive self assessment of their mental health versus 49% for assessments of their overall mood. By contrast there was negligible movement among liberals. This finding would be consistent with the possibility that conservatives inflate self-assessments of their mental health due to the stigmatization of the term. However, we cannot rule out the possibility that there is a real gap between mental health and overall mood ratings among conservatives but no such gap among liberals.

On the negative side of the scale, we see an opposite pattern. Here, the share of conservatives providing assessments of “fair” or “poor” is only modestly different (and not statistically significant). By contrast, the percent of liberals providing a negative assessment of their overall mood (17%) is about 9 points less than the rate of negative self assessment of their mental health (26%). This difference is statistically significant (p = .049).

**Fig 3:**
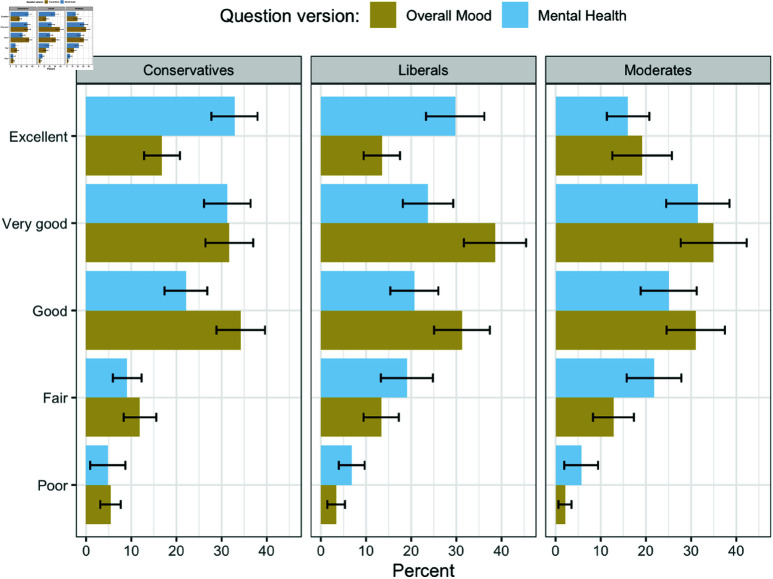
Distribution of self-assessments for Conservatives, Moderates, and Liberals based on experimental condition.

Figure 3 provides more detail on how the experimental wording treatment influenced responses for different ideological groups. Both conservatives and liberals were significantly less likely to rate their overall mood as excellent as compared to their mental health. However, for liberals, there was a significant increase in the propensity to provide a “very good” rating of their overall mood compared to their mental health, whereas conservatives were equally likely to use the “very good” response regardless of whether the mental health or overall mood terminology was used. Instead, conservatives were significantly more likely to rate their overall mood as merely good than they were to do the same for their mental health.

On the low end of the scale, there were no significant differences in the propensity of conservatives to respond with “poor” or “fair” depending on the terminology used. However, liberals were significantly more likely to say their mental health was poor than they were to assess their overall mood as poor.

The final panel in the figure shows the distribution of responses among those we identify as moderates – respondents who selected either “middle of the road” or “not sure” when asked to rate their own ideology. Similar to liberals, these individuals are more likely to provide negative ratings of their mental health than they are for their overall mood.

## Discussion

In news headlines and in a significant body of scholarship, much attention has been paid to the notion that conservatives have better mental health than liberals [1, 2, 4, 5, 7–13, 15, 19, 20, 23, 52–54]. The narrative that has emerged holds that the left is perpetually unhappy and depressed, while conservatives are in high spirits. In line with much of this research, we find that even after accounting for a variety of other factors there is a clear propensity of conservatives to provide more positive assessments of their mental health in comparison to liberals. This difference could be important if it reflects real differences in levels of depression, substance abuse, or suicide. Indeed, if conservatives are living more fruitful lives without mental health concerns because of their ideology, then we ought to pay attention to this divide and its political implications.

However, when thinking about any sort of "well-being gap" between liberals and conservatives, using different measures of self-assessed well-being may lead us to different conclusions. Using the term "mental health" may suggest that the well-being gap does indeed exist, but as we show in Study 2, asking instead about "overall mood" elimates such a gap.

While conservatives report significantly better mental health than liberals, there is no difference between how the two sides report their overall mood. Only conservatives give significantly different responses when prompted with the “mental health” terminology compared to “overall mood”. Mental health and overall mood are not perfect synonyms, thus it may be true that conservatives have better mental health than liberals, but perform no better than liberals when it comes to overall mood. If "mood" captures one’s short term well-being while mental health is longer term, then perhaps liberals and conservatives have no difference in short-term well-being, but conservatives are able to maintain that well-being for longer.

Another possibility is that it is the terminology itself that causes this gap in mental health ratings, but not overall mood ratings. Conservatives may view mental health as a sign of weakness, indicating their personal inability to manage their lives, despite their worldview’s belief in personal responsibility and control. They may view mental health as something dangerous to society [31, 32]. Indeed, one study finds that a theoretical political candidate diagnosed depression (as opposed to no diagnosis) suffered a significant drop in support from Republicans, but the difference was not significant with Democrats [34]. Or they may merely see the term mental health as something only the left cares about. In the former, conservative stigma surrounding mental health encourages inflated ratings to preserve the image of oneself. In the latter, self-inflating their mental health serves as a means to be dismissive of issues important to the left’s politics. In either case, in describing their own mental health, conservatives might want to reinforce a separation between themselves and those with mental illnesses.

In contrast, liberals and left-leaning groups are not prone to stigmatize mental health in this way [42, 51]. Instead, liberals were more likely to provide a negative assessment of their mental health than of their mood. Liberals may be more likely to provide negative assessments of mental health because of an increasing focus and awareness surrounding mental health issues and the term “mental health” in left-leaning spaces [55]. Left-leaning groups may actually encourage their members to identify with some sort of stigmatized identity, be it ethnic, racial, or a person with a physical or mental disability [45, 46]. Information about mental illnesses is also often spread on left-leaning social media spaces. Articles or clips with titles such as “signs you might have BPD” might prompt liberals to identify with having a mental illness [56]. Both the moral culture of left spaces, and an increasing focus on “mental health” in the media may explain why liberals may be more likely to identify with having a mental illness, or seek out care even when their symptoms are fairly low or moderate [20].

Ultimately, the results from our experiment suggest that using different measures of “well-being” may lead to different conclusions about whether or not conservatives are truly “happier” than liberals. The story surrounding whether or not the “happiness gap" exists may depend on which term you use as a proxy for “happiness." It is worth understanding both the subtle differences that these terms convey, as well as how people might respond to these terms in different ways. Given the significant news coverage that these findings receive in the mainstream media, it is especially important to emphasize that the existence of this ideological well-being gap appears to depend on the terminology one uses when asking Americans to provide these self-assessments.

## Supporting information

S1 Appendix Full question wording for variables used in analysis. (DOCX)

S1 Table Regression results behind Fig. 1. The following table presents OLS coefficients with standard errors in parentheses. Starred coefficients are significant at p < .01.
(PDF)

S2 Table Regression results for Study 1 when combining most positive and most negative response categories. The following table presents OLS coefficients with standard errors in parentheses. Starred coefficients are significant at p < .01.
(PDF)

S3 Table Regression results for study 1 when using issue positions to measure respondent ideology. The following table presents OLS coefficients with standard errors in parentheses. Starred coefficients are significant at p < .01. The ideology variable in these models is the percentage of conservative positions taken on issue questions rather than ideological self-identification.
(PDF)

S4 Table Robustness of results for study 2 when controlling for age. The following table presents OLS coefficients with standard errors in parentheses. Starred coefficients are significant at p < .01. The first model shows the conditional effect of seeing the term “mental health” rather than “overall mood” conditional on a respondent’s ideological self-identification. The second model includes the same conditional effects in addition to accounting for the effect of the treatment conditional on the respondent’s age.
(PDF)
