# Supplementary material for: Do conservatives really have better mental well-being than liberals?
Source: PLoS One. 2025 Apr 30;20(4):e0321573. doi: 10.1371/journal.pone.0321573 (PMC12043138; doi:10.1371/journal.pone.0321573)
Supplement: S1 Appendix — (DOCX) [file pone.0321573.s001.docx]

Mental health: Would you say that in general your mental health is...

(Allows one selection)

[1] Excellent

[2] Very good

[3] Good

[4] Fair

[5] Poor

Mood: Would you say that in general your overall mood is...

(Allows one selection)

[1] Excellent

[2] Very good

[3] Good

[4] Fair

[5] Poor

Ideology: How would you rate each of the following individuals and groups? Yourself

[1] Very Liberal

[2] Liberal

[3] Somewhat Liberal

[4] Middle of the Road

[5] Somewhat Conservative

[6] Conservative

[7] Very Conservative

[8] Not sure

Age: In what year were you born?

Education: What is the highest level of education you have completed?

(Allows one selection)

[1] Did not graduate from high school

[2] High school graduate

[3] Some college, but no degree (yet)

[4] 2-year college degree

[5] 4-year college degree

[6] Postgraduate degree (MA, MBA, MD, JD, PhD, etc.)

Race: What racial or ethnic group best describes you?

(Allows one selection)

[1] White

[2] Black or African-American

[3] Hispanic or Latino

[4] Asian or Asian-American

[5] Native American

[8] Middle Eastern

[6] Two or more races

[7] Other

Social media: In the past 24 hours have you... (check all that apply)

(Allows multiple selections)

[1] Used social media (such as Facebook or Youtube)

[2] Watched TV news

[3] Read a newspaper in print or online

[4] Listened to a radio news program or talk radio

[5] None of these

Recent life experiences: Over the past year have you...

(Allows multiple selections)

[1] Married

[2] Lost a job

[3] Finished school

[4] Retired

[5] Divorced

[6] Had a child

[7] Taken a new job

[9] Been a victim of a crime

[10] Visited an emergency room

[11] Visited a doctor for a regular examination

[12] Received a raise at work

[13] Had a pay cut at work

Covid-19: Have you or someone you know been diagnosed with the novel coronavirus (COVID-19) during the past year? (select all that apply)

(Allows multiple selections)

[1] Yes, I have

[2] Yes, a family member

[3] Yes, a friend

[4] Yes, a co-worker

[5] No, I do not know anyone who has been diagnosed

Vaccinated: Which of the following best describes you when it comes to being vaccinated against COVID-19?

(Allows one selection)

[1] I am fully vaccinated and have received at least one booster shot

[2] I am fully vaccinated but have not received a booster shot

[3] I am partially vaccinated (I have received the first of two shots for either Pfizer or Moderna)

[4] I am not vaccinated at all

Moved: How long have you lived at your present address?

(Allows one selection)

[1] Less than 1 month

[2] 2 to 6 months

[3] 7 to 11 months

[4] 1 to 2 years

[5] 3 to 4 years

[6] 5 or more years

Community type: How would you describe the place where you live?

(Allows one selection)

[1] City

[2] Suburb

[3] Town

[4] Rural area

[5] Other

Employment: Which of the following best describes your current employment status?

(Allows one selection)

[1] Working full time now

[2] Working part time now

[3] Temporarily laid off

[4] Unemployed

[5] Retired

[6] Permanently disabled

[7] Taking care of home or family

[8] Student

[9] Other

Church attendance: Aside from weddings and funerals, how often do you attend religious services?

(Allows one selection)

[1] More than once a week

[2] Once a week

[3] Once or twice a month

[4] A few times a year

[5] Seldom

[6] Never

[7] Don't know

Marital status: What is your marital status?

(Allows one selection)

[1] Married

[2] Separated

[3] Divorced

[4] Widowed

[5] Never married

[6] Domestic / civil partnership

Home owner: Do you own your home or pay rent?

(Allows one selection)

[1] Own

[2] Rent

[3] Other

Follow politics: Some people seem to follow what's going on in government and public affairs most of the time, whether there's an election going on or not. Others aren't that interested. Would you say you follow what's going on in government and public affairs ...

(Allows one selection)

[1] Most of the time

[2] Some of the time

[3] Only now and then

[4] Hardly at all

[7] Don't know

Income: Thinking back over the last year, what was your family's annual income?

(Allows one selection)

[1] Less than \$10,000

[2] \$10,000 - \$19,999

[3] \$20,000 - \$29,999

[4] \$30,000 - \$39,999

[5] \$40,000 - \$49,999

[6] \$50,000 - \$59,999

[7] \$60,000 - \$69,999

[8] \$70,000 - \$79,999

[9] \$80,000 - \$99,999

[10] \$100,000 - \$119,999

[11] \$120,000 - \$149,999

[12] \$150,000 - \$199,999

[13] \$200,000 - \$249,999

[14] \$250,000 - \$349,999

[15] \$350,000 - \$499,999

[16] \$500,000 or more

[97] Prefer not to say

Parent: Are you the parent or guardian of any children under the age of 18?

(Allows one selection)

[1] Yes

[2] No
