# Supplementary material for: Do conservatives really have better mental well-being than liberals?
Source: PLoS One. 2025 Apr 30;20(4):e0321573. doi: 10.1371/journal.pone.0321573 (PMC12043138; doi:10.1371/journal.pone.0321573)
Supplement: S1 Table — The following table presents OLS coefficients with standard errors in parentheses. Starred coefficients are significant at p < .01. (PDF) [file pone.0321573.s002.pdf]

	Model	
	(1)	(2)
Ideology	0.189* (0.004)	0.114* (0.004)
Got married in the last year		0.054* (0.006)
Got divorced		−0.020 (0.010)
Lost job		−0.025* (0.004)
Got a new job		−0.008 (0.003)
Got a pay raise		0.008* (0.003)
Had pay cut		0.007 (0.005)
Retired		0.0003 (0.006)
Currently unemployed		−0.027* (0.004)
Finished school		0.032* (0.006)
Had a child		0.010 (0.007)
Victim of a crime		−0.066* (0.005)
Went to the ER		−0.051* (0.003)
Had a doctor's visit		−0.026* (0.002)
Vaccinated for Covid-19		−0.022* (0.003)
Have had Covid-19		−0.026* (0.002)
Live in City		0.036* (0.003)
Live in suburbs		0.011* (0.003)
Moved in past year		−0.009 (0.003)
College degree		0.013* (0.003)
Attend church at least monthly		0.065* (0.002)
Income under $40k		−0.031* (0.004)
Income $40k-100k		0.008 (0.004)
Income over $100k		0.038* (0.005)
Home owner		0.024* (0.003)
Own stocks		0.023* (0.003)
Can't pay $400 expense		−0.054* (0.002)
Social media user		−0.022* (0.003)
White		−0.020* (0.003)
Black		0.057* (0.004)
Have a child under 18 years old		0.023* (0.003)
Follow politics most of the time		0.042* (0.002)
Age		−0.0003 (0.0004)
Age squared		0.00004* (0.00000)
Is married		0.021* (0.003)
Constant	0.488* (0.002)	0.456* (0.011)
Observations	59,668	59,033
R^2	0.038	0.204
Adjusted R^2	0.038	0.204
