# Supplementary material for: Do conservatives really have better mental well-being than liberals?
Source: PLoS One. 2025 Apr 30;20(4):e0321573. doi: 10.1371/journal.pone.0321573 (PMC12043138; doi:10.1371/journal.pone.0321573)
Supplement: S2 Table — The following table presents OLS coefficients with standard errors in parentheses. Starred coefficients are significant at p < .01. (PDF) [file pone.0321573.s003.pdf]

	Dependent variable:			
	Excellent or very good		Fair or poor	
	(1)	(2)	(3)	(4)
Ideology	0.275* (0.007)	0.157* (0.007)	−0.215* (0.006)	−0.121* (0.006)
Got married in the last year		0.074* (0.010)		−0.048* (0.009)
Got divorced		−0.034 (0.018)		0.033 (0.015)
Lost job		−0.015 (0.007)		0.048* (0.006)
Got a new job		−0.029* (0.006)		−0.004 (0.005)
Got a pay raise		0.002 (0.005)		−0.021* (0.005)
Had pay cut		0.014 (0.010)		−0.0004 (0.008)
Retired		−0.012 (0.011)		−0.012 (0.009)
Currently unemployed		−0.036* (0.007)		0.034* (0.006)
Finished school		0.051* (0.011)		−0.026* (0.009)
Had a child		0.004 (0.012)		−0.020 (0.010)
Victim of a crime		−0.061* (0.009)		0.100* (0.008)
Went to the ER		−0.073* (0.005)		0.071* (0.004)
Had a doctor's visit		−0.038* (0.004)		0.030* (0.004)
Vaccinated for Covid-19		−0.041* (0.005)		0.009 (0.004)
Have had Covid-19		−0.034* (0.004)		0.030* (0.004)
Live in City		0.047* (0.005)		−0.045* (0.004)
Live in suburbs		0.017* (0.005)		−0.024* (0.004)
Moved in past year		−0.014 (0.006)		0.007 (0.005)
College degree		0.003 (0.005)		−0.023* (0.004)
Attend church at least monthly		0.107* (0.004)		−0.072* (0.004)
Income under $40k		−0.038* (0.007)		0.050* (0.006)
Income $40k-100k		0.015 (0.007)		−0.007 (0.006)
Income over $100k		0.068* (0.008)		−0.027* (0.007)
Home owner		0.030* (0.005)		−0.035* (0.004)
Own stocks		0.043* (0.005)		−0.031* (0.004)
Can't pay $400 expense		−0.085* (0.004)		0.068* (0.004)
Social media user		−0.026* (0.005)		0.034* (0.004)
White		−0.032* (0.005)		0.013* (0.005)
Black		0.093* (0.007)		−0.064* (0.006)
Have a child under 18 years old		0.022* (0.005)		−0.042* (0.005)
Follow politics most of the time		0.072* (0.004)		−0.017* (0.004)
Age		−0.004* (0.001)		−0.004* (0.001)
Age squared		0.0001* (0.00001)		−0.00001 (0.00001)
Is married		0.037* (0.005)		−0.027* (0.004)
Constant	0.326* (0.004)	0.352* (0.018)	0.359* (0.003)	0.507* (0.016)
Observations	59,668	59,033	59,668	59,033
R^2	0.028	0.153	0.023	0.152
Adjusted R^2	0.028	0.152	0.023	0.151
