# Supplementary material for: Do conservatives really have better mental well-being than liberals?
Source: PLoS One. 2025 Apr 30;20(4):e0321573. doi: 10.1371/journal.pone.0321573 (PMC12043138; doi:10.1371/journal.pone.0321573)
Supplement: S3 Table — The following table presents OLS coefficients with standard errors in parentheses. Starred coefficients are significant at p < .01. The ideology variable in these models is the percentage of conservative positions taken on issue questions rather than ideological self-identification. (PDF) [file pone.0321573.s004.pdf]

	Model:	
	(1)	(2)
Ideology (issues)	0.267* (0.005)	0.189* (0.005)
Got married in the last year		0.052* (0.006)
Got divorced		−0.022 (0.010)
Lost job		−0.024* (0.004)
Got a new job		−0.006 (0.003)
Got a pay raise		0.010* (0.003)
Had pay cut		0.001 (0.005)
Retired		−0.00005 (0.006)
Currently unemployed		−0.028* (0.004)
Finished school		0.030* (0.006)
Had a child		0.005 (0.007)
Victim of a crime		−0.067* (0.005)
Went to the ER		−0.051* (0.003)
Had a doctor's visit		−0.024* (0.002)
Vaccinated for Covid-19		−0.007 (0.003)
Have had Covid-19		−0.025* (0.002)
Live in City		0.038* (0.003)
Live in suburbs		0.014* (0.003)
Moved in past year		−0.010* (0.003)
College degree		0.016* (0.003)
Attend church at least monthly		0.059* (0.002)
Income under $40k$		−0.027* (0.004)
Income $40k - 100k$		0.012* (0.004)
Income over $100k$		0.041* (0.005)
Home owner		0.020* (0.003)
Own stocks		0.022* (0.003)
Can't pay $400expense$		−0.053* (0.002)
Social media user		−0.018* (0.003)
White		−0.023* (0.003)
Black		0.060* (0.004)
Have a child under 18 years old		0.023* (0.003)
Follow politics most of the time		0.038* (0.002)
Age		−0.001 (0.0004)
Age squared		0.00004* (0.00000)
Is married		0.019* (0.003)
Constant	0.481* (0.002)	0.434* (0.010)
Observations	59,710	59,074
R^2	0.052	0.213
Adjusted R^2	0.052	0.212
