# Supplementary material for: Do conservatives really have better mental well-being than liberals?
Source: PLoS One. 2025 Apr 30;20(4):e0321573. doi: 10.1371/journal.pone.0321573 (PMC12043138; doi:10.1371/journal.pone.0321573)
Supplement: S4 Table — The following table presents OLS coefficients with standard errors in parentheses. Starred coefficients are significant at p < .01. The first model shows the conditional effect of seeing the term “mental health” rather than “overall mood” conditional on a respondent’s ideological self-identification. The second model includes the same conditional effects in addition to accounting for the effect of the treatment conditional on the respondent’s age. (PDF) [file pone.0321573.s005.pdf]

	Model	
	(1)	(2)
Mental Health wording	−0.065 (0.031)	−0.197* (0.056)
Ideology: Liberal	−0.027 (0.031)	−0.029 (0.031)
Ideology: Conservative	−0.034 (0.030)	−0.031 (0.030)
Age		−0.001 (0.001)
Mental Health wording × Liberal	0.077 (0.044)	0.081 (0.044)
Mental Health wording × Conservative	0.154* (0.042)	0.134* (0.043)
Mental Health wording × Age		0.003* (0.001)
Constant	0.641* (0.021)	0.689* (0.039)
Observations	999	999
R^2	0.018	0.027
Adjusted R^2	0.013	0.020
